# Oil Infrastructure has Greater Impact than Noise on Stress and Habitat Selection in Three Grassland Songbirds

**DOI:** 10.1007/s00267-022-01752-2

**Published:** 2022-12-02

**Authors:** Paulson Given Des Brisay, Laura Diane Burns, Kevin Ellison, William Gary Anderson, Marty Leonard, Nicola Koper

**Affiliations:** 1 Natural Resources Institute, University of Manitoba, Winnipeg, MB Canada; 2 Northern Great Plains Program, American Bird Conservancy, Bozeman, MT USA; 3 Department of Biology, University of Manitoba, Winnipeg, MB Canada; 4 Department of Biology, Dalhousie University, Halifax, MB Canada; 5 Present Address: Canadian Wildlife Service, Environment and Climate Change Canada, Winnipeg, MB Canada; 6 Present Address: Conservation and Research Department, Assiniboine Park Zoo, Winnipeg, MB Canada

**Keywords:** Energy development, Anthropogenic noise, Habitat quality, Stress, Grassland birds, Conservation

## Abstract

Oil extraction may impact wildlife by altering habitat suitability and affecting stress levels and behavior of individuals, but it can be challenging to disentangle the impacts of infrastructure itself on wildlife from associated noise and human activity at well sites. We evaluated whether the demographic distribution and corticosterone levels of three grassland passerine species (Chestnut-collared Longspur, *Calcarius ornatus*; Baird’s Sparrow, *Centronyx bairdii*; and Savannah Sparrow *Passerculus sandwichensis*) were impacted by oil development in southern Alberta, Canada. We used a landscape-scale oil well noise-playback experiment to evaluate whether impacts of wells were caused by noise. Surprisingly, higher-quality female Chestnut-collared Longspurs tended to nest closer to oil wells, while higher-quality Savannah Sparrows generally avoided nesting sites impacted by oil wells. Corticosterone levels in all species varied with the presence of oil development (oil wells, noise, or roads), but the magnitude and direction of the response was species and stimulus specific. While we detected numerous impacts of physical infrastructure on stress physiology and spatial demographic patterns, few of these resulted from noise. However, all three species in this study responded to at least one disturbance associated with oil development, so to conserve the grassland songbird community, both the presence of physical infrastructure and anthropogenic noise should be mitigated.

## Introduction

Oil and gas development across the North American Great Plains has resulted in the conversion of more than 1.5 million hectares of grassland to well pads, roads, and storage facilities (Allred et al. 2015). This constitutes approximately 1% of all remaining intact grassland in the Great Plains (World Wildlife Fund 2021). However, despite this relatively small footprint, infrastructure may degrade much larger areas of adjacent habitat through edge effects and chronic noise (Sliwinski and Koper 2012; Francis and Barber 2013). In Canada, severe habitat losses from conversion of native grasslands to croplands in the last century have left the Central Mixed-grass Prairie and Norther Fescue Mixed-grass Prairie qualifying as Endangered according to the IUCN’s criteria for the Redlist of Ecosystems (Gage et al. 2016; Comer et al. 2018). Consequently, the majority of grassland songbird species have experienced range-wide population declines since 1966 (Sauer et al. 2017; Rosenberg et al. 2019). While land conversion to crop agriculture was likely the primary driver of these declines and crop and biofuel production remains a significant driver of grassland loss (Lark et al. 2015; Shaffer et al. 2019), habitat conversion and degradation associated with oil and gas development has also recently become a significant threat to the integrity of remaining habitat for grassland birds (Shaffer et al. 2019). While increasingly, research has shown grassland birds avoid areas affected by energy development (Nenninger and Koper 2018), or suffer demographic consequences from nesting in affected habitat (Bernath-Plaisted and Koper 2016), relatively few studies have been able to experimentally identify the precise mechanisms driving these observations (Rosa et al. 2015). Understanding such mechanisms is critical to the conservation and management of grassland birds, as different management solutions may be required to mitigate different kinds of effects (Francis and Barber 2013).

Construction, industrial activities, infrastructure, and vehicle traffic associated with oil development can cause direct mortality of grassland songbirds (Northrup and Wittemyer 2013), or indirectly increase their predation risk (Andersson et al. 2009; Campos et al. 2009). Noise from oil extraction can simultaneously decrease an individual’s ability to detect predators or identify the presence of conspecifics. For example, energetic masking of cues used to detect predators (Slabbekoorn and Ripmeester 2008) or conspecific alarm calls (Antze and Koper 2018) may increase chances of nest predation, or increase stress and thus alter corticosterone levels (Kleist et al. 2018). Noise might create an ecological trap (Robertson and Hutto 2006) if it prevents birds from detecting predators in the area, but the opposite consequence is also possible; acoustically sensitive species may avoid noisy areas, even if oil development has a neutral or positive impact on reproductive output by decreasing predator abundance or activity (Francis et al. 2009; Lendrum et al. 2017), or decreasing resource competition (Francis et al. 2012).

Because anthropogenic noise normally co-occurs with the presence of the physical infrastructure producing it, it can be challenging to determine whether noise is responsible for observed impacts of the built environment, and this becomes a problem when attempting to develop effective management solutions to problems resulting from anthropogenic activities. A landscape-scale noise-playback experiment has demonstrated that traffic noise per se results in decreased mass gain of birds during migration (Ware et al. 2015); conversely, nesting success can actually increase as compressor station noise increases when nest predators avoid noise (Francis et al. 2009). However, other research on energy development has suggested that noise plays a minimal role in explaining its impacts on grassland birds; for example, negative impacts of oil wells on abundance (Nenninger and Koper 2018) and nesting success (Bernath-Plaisted and Koper 2016) of grassland passerines did not decrease when wells were turned off and thus were silent. Nonetheless, the mechanisms that explain impacts of wells on abundance (site avoidance) and nesting success (predator density and activity) differ from mechanisms that impact health of adults and nestlings (e.g., physiological stress), so further research is necessary to fully understand impacts of noise per se from oil development on grassland birds.

One mechanism by which species may respond to differences in habitat quality is by differential selection. However, such processes may be mediated by demographics or variation in individual quality. In such cases, differences in perceived habitat quality may not be readily apparent in studies of abundance or occupancy. Conversely, in demographic studies, effects driven by individual quality and habitat preference may be wrongly attributed to other sources. The ideal despotic distribution model (Fretwell 1972) suggests that high quality, or competitive, birds should choose the best habitat in which to breed and exclude lower quality, or less competitive, individuals from those breeding sites. The Great Plains are a spatially and temporally heterogeneous landscape due to both human disturbance and natural variation. Additionally, philopatry among migrant grassland passerines is generally low (Jones et al. 2007), possibly due to this high temporal unpredictability in grassland patch productivity (Doligez et al. 2003). Thus, selection should be particularly strong for high quality individuals to accurately assess habitat quality at breeding sites between years. Individual quality can be independently assessed using metrics of productivity, such as clutch sizes or nestling quality (Holmes et al. 1996). Alternatively, young, naive breeders lack the experience or social status to select or defend high quality breeding sites (Holmes et al. 1996; Habib et al. 2007). Smaller individuals may also lack the competitive ability to defend prime territories or acquire high-quality mates (Linhart and Fuchs 2015). Therefore, if there is a high proportion of younger, smaller, or less productive individuals in an area, that area is likely perceived as low-quality habitat by dominant individuals.

Individuals that breed in lower quality habitat may suffer direct fitness costs including reduced nesting success (Lloyd and Martin 2005; Bernath-Plaisted and Koper 2016), reduced nestling quality (Potvin and MacDougall-Shackleton 2015), or physiological stress (McEwen and Wingfield 2003; Busch and Hayward 2009). In birds, the primary hormone involved in mediating the stress response is corticosterone, a glucocorticoid released from the Hypothalmic-Pituitary-Adrenal (HPA) axis (Wingfield et al. 1992; Romero et al. 2009). Baseline stress levels can be approximated by measuring circulating levels of corticosterone under normal allostatic conditions, and the magnitude of a stress response can be measured as the increase in circulating levels of corticosterone in response to a stressor (Wingfield et al. 1998). Predation risk (real or perceived), human disturbance (visual or auditory), and habitat degradation (e.g. invasive species, toxicants) may all lead to increased physiological stress (Boonstra et al. 1998; Newcomb Homan et al. 2003; Maron et al. 2012). In combination, the measures of basal corticosterone and stress response can be useful indicators of an individual’s ability to cope with its current environment and could be used to identify individuals living in sub-optimal conditions (Kleist et al. 2018).

Here we used a landscape-scale manipulative experiment to evaluate the impacts of oil development and associated noise on the demographic distribution and corticosterone levels of three grassland songbirds found across the Northern Great Plains of North America: Chestnut-collared Longspur (*Calcarius ornatus*), Baird’s Sparrow (*Centronyx bairdii*), and Savannah Sparrow (*Passerculus sandwichensis*). This design enabled us to directly assess the sublethal effects of oil development on grassland bird populations and determine if anthropogenic noise is their primary driver. Our focal species were selected to encompass the range of habitat preferences, population trends, Species at Risk listings, and sensitivities to energy development typical of this grassland songbird community. Chestnut-collared Longspurs are mixed-grass prairie obligates that are Endangered in Canada (COSEWIC 2019) and Birds of Management Concern in the USA (Somershoe 2018). This species is area and edge-sensitive (Davis 2004; Sliwinski and Koper 2012) has lower nesting success in the presence of exotic vegetation (Lloyd and Martin 2005), but surprisingly, recent studies have reported few effects of oil and gas wells on their abundance or productivity (Hamilton et al. 2011; Bernath-Plaisted and Koper 2016; Yoo and Koper 2017; Rodgers and Koper 2017; Nenninger and Koper 2018). Baird’s Sparrow is a species of Special Concern in Canada (COSEWIC 2012) and Management Concern in the U.S. (Somershoe 2018), and avoids oil wells (Nenninger and Koper 2018). Savannah Sparrows are grassland generalists not listed as species at risk in Canada or the U.S. In southeastern Alberta the presence of oil development does not negatively impact abundance (Nenninger and Koper 2018), but their nesting success is lower near oil wells (Bernath-Plaisted and Koper 2016).

## Methods

This research was conducted under University of Manitoba animal care protocol F15-005, Canadian bird banding permit 10840(A), Canadian Wildlife Service permit #11-MB/SKL/AB-SC007, and Alberta Environment and Sustainable Research Development Research Permit #56016 and Collection License #56017.

For further details regarding methods, see Supplemental Information.

### Study Design

During the breeding seasons (May 1 to July 31) of 2015 to 2017, we studied Chestnut-collared Longspurs, Baird’s Sparrows, and Savannah Sparrows within 60 km of Brooks, Alberta, Canada (50°33′51″N 111°53′56″W, 760 MASL) on land owned by the Eastern Irrigation District, a private landowner that primarily uses grasslands for grazing cattle (*Bos taurus*). Grazing activities varied by year and by site based on the operational needs of the EID, but not in a structured way with any of the treatment types in this study. Our study sites were native mixed-grass prairies intermixed with a very low abundance of invasive grasses and forbs. This region is relatively arid, receiving an average of 252.6 mm of rainfall annually (Environment and Climate Change Canada 2017). Pastures were divided by three-wire fences into Sections (1.6 × 1.6 km) or larger, which are accessed by gravel, or occasionally paved, roads. Active lease sites (~1 ha containing 1–3 well heads on 50 × 50 m of level gravel) are operated for extracting subsurface oil and natural gas in this region. We established study sites where all three focal songbird species were confirmed to be present, ensuring that site-scale vegetation was suitable for each study species and was similar among treatments.

Study sites were centered on either an active oil well, a playback unit with or without simulated oil well noise, or were control sites with neither oil wells nor noise-playback units. Oil well sites contained one of two commonly used pump types that differ in their physical size and the noise spectra and power they produce (Warrington et al. 2018). Oil wells are visited by oil workers on a semi-regular basis (every few days) via dirt or gravel service roads, which comprises the majority of the traffic on the roads in our study area. Screwpumps (*n* = 4, *h* = 2.7 m, ~80–90 dB(Z) at <10 m) were shorter, moved less, and produced higher sound pressure levels than pumpjacks (*n* = 3, *h* = 4.5 m, ~55–80 dB(Z) at <10 m). All oil wells were generator-powered (pump mechanism powered by a generator) and produce cyclic, predictable broad-band noise 24 h per day (Warrington et al. 2018). To distinguish the effects of noise from effects of the presence of physical infrastructure, in 2016 and 2017 we added sites with high-fidelity playback units (*n* = 6) broadcasting recordings of generator-powered screwpumps (simulated screwpump noise) 24 h a day prior to territory establishment and throughout the breeding season (approx. May 1 to July 31). These units accurately mimic the spectral composition, sound pressure levels, and frequencies of real infrastructure noise (see Rosa et al. 2015a; Rosa and Koper 2018). Each playback unit included a solar panel array, small housing unit for batteries, 8 GB iPod Nanos (Apple Inc., Cupertino, CA) playing WAV files, two high-fidelity speakers facing in opposite directions, with total dimensions of 5 m (l) × 1.5 m (w) × 1.5 m (h), and were surrounded by a ~1-m tall metal fence to prevent cattle from accessing the equipment. We also established silent playback sites (*n* = 6) with identical equipment as the playback units, but without broadcasting noise, to control for the potential impacts of the playback equipment itself on habitat selection or quality. To ensure no overlap with, other large or noisy nearby disturbances, each site center was a minimum of 800 m from the next nearest site center or active oil well. This effectively limits the cumulative effects of noise for any given bird since the majority of the real or simulated oil well noise in our study has attenuated within that distance (Rosa et al. 2015). Additionally, playback, silent, and control site centers were a minimum of 400 m from the nearest road to control for both the physical presence of infrastructure and the associated industrial activity.

### Field Methods

We captured adult birds to determine age as Second Year (SY) or After Second Year (ASY) (Pyle et al. 2008), sex, mass, and tarsus length. We used target nets (30 mm mesh mist nets surrounding a painted model bird and speaker) for actively displaying males or walk-in drop traps (mist netting material over a wire frame propped over a nest) for nesting pairs actively feeding 3–10 day old nestlings. All individuals were banded with a unique numbered Canadian Wildlife Service metal band and a unique combination of two or three colored plastic bands (Darvic) for subsequent identification in the field. For any captured adults with a known nest location, we measured nestling mass and defined brood size as the number of young in the nest. For all species, we measured the nestlings when they were 6–8 days old, or on the day they were found for nests containing older nestlings (age range: 6–11 days, mean age: 7.98 days). Nestling age was assessed based on hatch date (when known) and visual characteristics including primary pin length and unsheathing (Jongsomjit et al. 2007).

To determine baseline levels of circulating corticosterone (hereafter “basal cort”) and the capacity of an individual to respond to a novel stressor (change in corticosterone level during handling; hereafter “stress response”) we collected two < 70 µL blood samples via brachial venipuncture using heparinized micro-capillary tubes. The first sample was collected within three minutes of capture to represent basal cort (Romero and Reed 2005) and the second sample was collected after 12 min post capture following a standardized stress handling protocol (Wingfield et al. 1992; Lynn et al. 2003). The stress response was calculated as the difference between the first and second samples. Blood samples were collected and kept on ice (<6 h) until the plasma was separated and frozen at −20 °C until extraction. Plasma corticosterone concentrations were determined using a radioimmunoassay after extraction using absolute ethanol. For assay details see the Supplemental Information.

### Landscape Characterization

We mapped all oil wells and range roads within 1000 m of all territories (nest locations, if known, or capture location) using handheld GPS units (Garmin etrex 20) and determined the locations of oil wells beyond that limit from a GIS layer of all active oil lease sites in our study region. We measured the minimum distance from each territory to the nearest oil well, road, and experimental treatment (screwpump, pumpjack, simulated screwpump noise, silent playback, or the center of the control site).

### Statistical Analyses

All analyses were conducted separately by species and sex. We used null hypothesis significance testing (Mundry 2011) and an alpha level of 0.1 to determine the statistical significance of the effects of oil development and noise, as is often used in conservation when the consequences of a Type II error outweigh those of a Type I error (Taylor and Gerrodette 1993). We conducted all statistical analyses in R (R Core Team 2019). We constructed generalized linear models and mixed models with the package LmerTest (Kuznetsova et al. 2017) to assess individual quality, and habitat selection and physiological responses to physical infrastructure and noise (Table 1). We used diagnostic graphs and deviance/df ratios to ensure we met assumptions of statistical tests.

**Table 1:** Generalized linear models and mixed models constructed in R with package LmerTest to assess individual quality (female characteristics associated with higher productivity at the nest scale), and habitat selection (age, size, weight) and physiological responses (basal cort and stress response) to physical infrastructure and noise ant the landscape and site scale

Scale	Response	Species	Model structure
Landscape	Age	All	dist. to oil well + dist. to road
	Size	All	dist. to oil well + dist. to road
	Weight	All	dist. to oil well + dist. to road + day
	Basal Cort	CCLO (F), SAVS (F)	dist. to oil well + dist. to road
		CCLO (M)	dist. to oil well + dist. to road + age
		SAVS (M)	dist. to oil well + dist. to road + age + day
		BAIS (M)	dist. to oil well + dist. to road + weight +day
	Stress Resp.	CCLO (M), SAVS (M)	dist. to oil well + dist. to road
		BAIS (M), CCLO (F)	dist. to oil well + dist. to road + time
		SAVS (F)	dist. to oil well + dist. to road + basal cort
Site	Age	All	dist. to site * treatment
	Size	All	dist. to site * treatment
	Weight	All	dist. to site * treatment + day
	Basal Cort	CCLO (F), SAVS (F)	dist. to site * treatment
		CCLO (M)	dist. to site * treatment + age
		SAVS (M)	dist. to site * treatment + age + day
		BAIS (M)	dist. to site * treatment + weight +day
	Stress Resp.	CCLO (M)	dist. to site * treatment
		BAIS (M), CCLO (F)	dist. to site * treatment + time
		SAVS (F)	dist. to site * treatment + basal cort
		SAVS (M)	dist. to site * treatment + basal cort + day
Nest	Nest Biomass	CCLO (F), SAVS (F)	F. age + F. weight + F. tarsus + brood size + N. Age
	Nestling Weight	CCLO (F), SAVS (F)	F. age + F. weight + F. tarsus + N. Age + (1|nest id)

*CCLO* Chestnut-collared Longspur, *BAIS* Baird’s Sparrow, *SAVS* Savannah Sparrow, *basal cort* baseline levels of circulating corticosterone, *stress resp* change in corticosterone level during handling, *dist. to oil well* distance to the nearest oil well, *dist. to road* distance to the nearest road, *day* ordinal day, *age* adult age as second year or after second year, *weight* bird weight at capture, *time* capture time, *brood size* number of nestling in nest at measuring, *N. age* age of nestling in days post hatch, *(1|nest id)* nest identification code as a random variable

We evaluated effects of oil infrastructure at two spatial scales: (1) to test for effects of roads and oil wells at a landscape scale, we included the minimum distance from each territory to the nearest oil well and the nearest road as continuous fixed effects. Correlation between these variables was low (*r* < 0.50). Information on well type was not available at this spatial scale. (2) To test for effects of well type and noise per se at the site scale (within 1000 m of the site center), we modelled treatment type (pumpjack, screwpump, simulated screwpump noise, silent playback, and control), distance to that experimental feature (or site center for controls), and their interactions as fixed effects. Fixed variables in models (1) and (2) were then included in most of the analyses as described below (Table 1). We tested whether the inclusion of year or site as random variables improved model fit for all preliminary models using AIC (Mundry 2011); this was not the case, so these random variables were not included in any final models.

We assessed whether adult age, mass, and size varied with distance to roads, wells, or noise. Fixed variables were those described above for the landscape (1) and site-scale (2) models (Table 1). Ordinal day was also included for all mass models to account for changes in mass across the breeding season. As no previous studies have evaluated what characteristics of Chestnut-collared Longspurs are correlated with quality of individuals, to allow us to interpret any spatial demographic distributions we detected, we also used our morphometric data to determine which physical characteristics of adults were indicative of higher productivity (more or heavier offspring; hereafter, “high-quality individuals”). We tested whether adult age (SY or ASY), mass (g), and size (tarsus length in mm) were correlated with nestling mass, brood biomass (mass of all nestlings combined). As not all nests were found during the laying or egg stage, we were not able to confidently analyze differences in brood size per se. Models for nestling mass included as fixed effects, adult age, mass, and size; nestling age; and nest ID as a random effect (Table 1). Models for brood biomass included adult age, mass, and size, brood size, and average nestling age as fixed effects (Table 1). In addition to productivity, we also explored whether larger or heavier males were social mates with higher quality females. We used the female traits that were significantly associated with productivity as dependent variables, and her socially paired male’s age, mass, and size as independent variables.

Before determining if there was an effect of oil-related disturbances on basal corticosterone and the stress response, we determined whether additional biologically important factors that might influence levels of corticosterone by species and sex should be included in our oil well and noise models. These variables included mass, age, day of season, and time of day as independent variables, and the stress response model also contained basal corticosterone as an independent variable. We then added any of these variables that were significant to the landscape (1) and site-scale (2) models described above (Table 1). We ran two linear models for each species-sex group; one with basal corticosterone as a response variable, and one with the stress response as the response variable.

## Results

We captured 456 adult birds and measured 214 nestlings (Table 2) between May 10th and July 23rd in 2015–2017 (mean capture date across years was June 10th). While all females were captured during the nestling stage, reproductive status was not known for all males. Our sample size of female Baird’s Sparrows and their associated nests were too small to analyze.

**Table 2:** Sample sizes by species and sex for adult and nesting birds handled during the 2015–2017 breeding seasons in southern Alberta

Species	Chestnut-collard Longspur		Baird’s Sparrow		Savanah Sparrow	
Sex	Female	Male	Female	Male	Female	Male
Birds captured	95	134	3	78	25	121
Basal cort	82	114	2	46	20	103
Stress response	79	110	2	45	19	101
Nests measured	40		0		12	
Nestings measured	178		0		36	

Basal cort (baseline levels of circulating corticosterone) and the stress response (change in corticosterone level during handling) represent the number of viable samples analyzed from those captured individuals

### Adult Quality

**Fig. 1:**
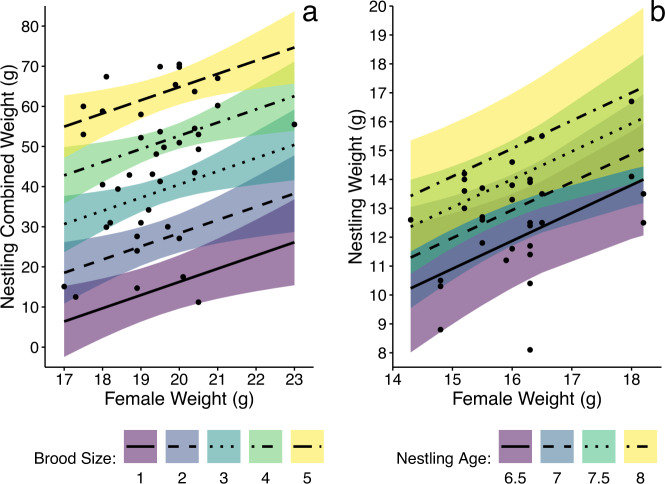
Heavier females are more productive. This is demonstrated by **a** higher combined nestling mass in Chestnut-collared Longspur and **b** heavier individual nestling mass in Savannah Sparrows. Lines represent the model predictions; shaded area represent the 90% confidence intervals for After Second Year females with a mean tarsus length; points are the raw data. All relationships shown are significant (*p* < 0.1)

Heavier females were more productive than lighter females for both Chestnut-collared Longspurs and Savannah Sparrows. After accounting for brood size, heavier female Chestnut-collared Longspurs produced broods with greater biomass, suggesting females were able to provide superior care evenly across all young in their broods (Fig. 1; detailed results Table S1). We infer that this was trend was driven by increased individual nestling weights, but that we did not have sufficient statistical power to detect this difference. Heavier female Savannah Sparrows had heavier individual nestlings after accounting for nestling age at banding, suggesting that their nestling grew faster than those of lighter females. Since their brood biomass was not significantly different than lighter females, we suspect that heavier females were putting more effort into raising fewer higher quality young (Fig. 1; detailed results Table S1). In both cases, this suggests that heavier females were of higher quality and lighter females were of lower quality. Male age, mass, and size were not correlated with productivity for either Chestnut-collared Longspurs or Savannah Sparrows (*p* > 0.10), nor were these physical characteristics correlated with the quality of their socially bonded female (*p* > 0.18).

### Spatial Demographic Patterns

**Fig. 2:**
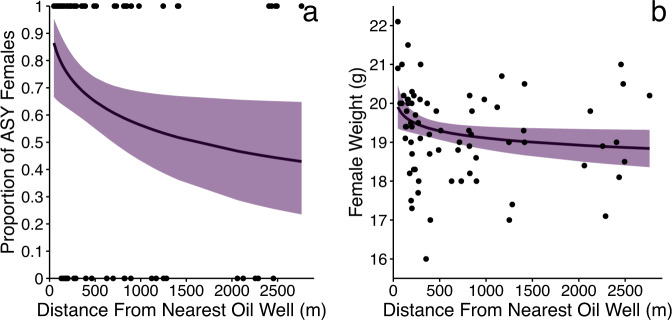
Female Chestnut-collared Longspur preferentially select habitat closer to oil wells. This is demonstrated by **a** females closer to oil wells are older, and **b** females closer to oil wells are heavier. Lines represent the model predictions; shaded area represent the 90% confidence intervals; points are the raw data. All relationships shown are significant (*p* < 0.1)

At the landscape scale (see detailed statistical results in Tables S2-4), female Chestnut-collared Longspurs near oil wells were older (*β* = −0.53, SE = 0.28, *p* = 0.06; Fig. 2) and heavier (*β* = −0.26, SE = 0.14, *p* = 0.06; Fig. 2) but Longspurs were smaller near roads (*β* = 0.15, SE = 0.7, *p* = 0.03). Female Savanah Sparrows did not show any spatial pattern in age, mass, or size relative to oil wells or roads. Similarly, we found no spatial pattern in age, mass, or size relative to oil wells or roads for males of any species. Our sample size of female Baird’s Sparrows was too small to analyze.

At the site scale (see detailed statistical results in Tables S5–S7), female Savannah Sparrows near screwpumps were smaller (*β* = 1.41, SE = 0.74, *p* = 0.074). Male Savannah Sparrows were lighter near pumpjacks (*β* = 0.83, SE = 0.37, *p* = 0.028) and near simulated screwpump noise (*β* = 0.89, SE = 0.49, *p* = 0.073), whereas male Baird’s Sparrows were heavier near screwpumps (*β* = −0.82, SE = 0.40, *p* = 0.044). Closer to silent playback infrastructure, female and male Chestnut-collared Longspurs and Baird’s Sparrows were all slightly heavier (*β* = −0.01, SE = 0.01, *p* < 0.032); we speculate that this result is not biologically meaningful, as the parameter estimate was extremely small in all cases. There were no significant spatial patterns in age for any species at the site scale.

### Corticosterone

Chestnut-collared Longspurs had lower basal corticosterone levels and smaller stress responses overall than either sparrow species (Fig. S1). Sex was a strong predictor of both basal corticosterone and magnitude of the stress response for all species, so for all analyses we treated male and females separately (Tables S8 and S9).

**Fig. 3:**
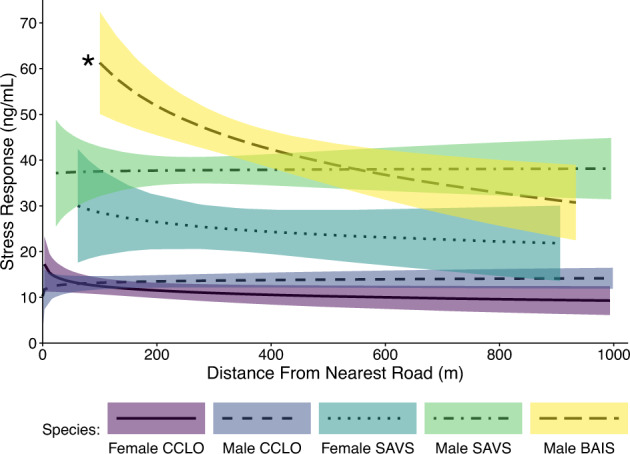
Stress response (increase in corticosterone levels during a stress handling protocol) relative to proximity to roads for all species; Baird’s Sparrows closer to roads had higher stress responses. (CCLO Chestnut-collared Longspur; BAIS Baird’s Sparrow; SAVS Savannah Sparrow). Lines represent the model predictions; shaded area represent the 90% confidence intervals; an asterisk indicates a significant interaction between distance and treatment (*p* < 0.1)

In several instances, birds living closer to oil wells, simulated screwpump noise, or roads had altered levels of corticosterone, though the influential stimulus and type of response was not consistent (statistical results Tables S10-13). In some cases, females had lower corticosterone levels near the physical oil infrastructure; female Savannah Sparrows had lower stress responses near oil wells at the landscape scale (*β* = 6.88, SE = 3.56, *p* = 0.07), and female Chestnut-collared Longspurs had lower basal corticosterone near pumpjacks (*β* = 0.92, SE = 0.54, *p* = 0.095; Fig. 3). This pattern differed from that observed near simulated well noise; at the site scale, female Chestnut-collared Longspurs had higher basal corticosterone levels near simulated screwpump noise (*β* = −0.88, SE = 0.36, *p* = 0.016; Fig. 3).

**Fig. 4:**
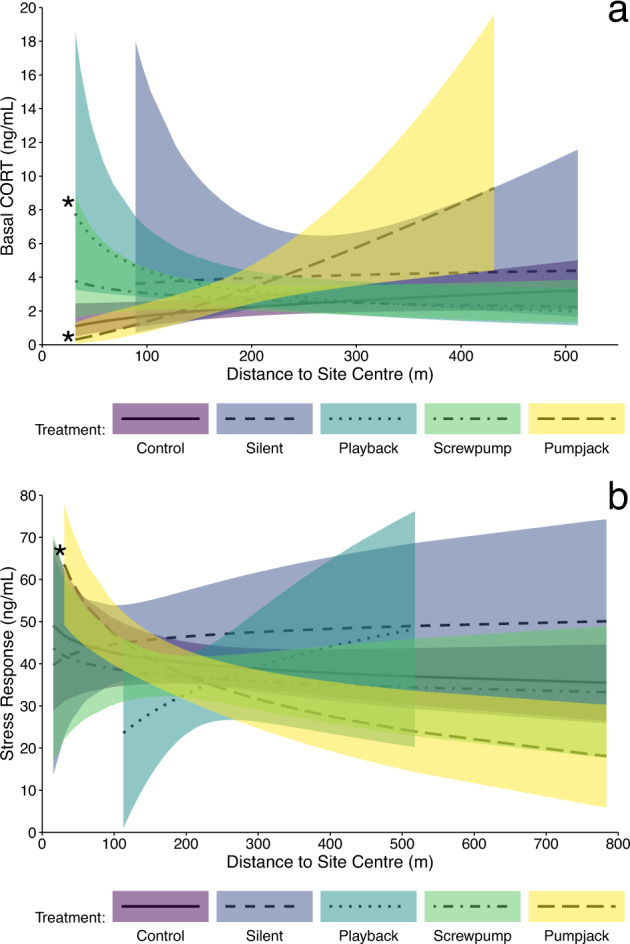
**a** Female Chestnut-collared Longspur basal corticosterone levels relative to proximity to treatment type; individuals closer to pumpjacks had lower basal corticosterone levels and individuals closer to simulated screwpump noise had higher basal corticosterone levels. **b** Male Savannah Sparrow stress response (increase in corticosterone levels during a stress handling protocol) relative to proximity to treatment type; individuals closer to pumpjacks has higher stress responses. (Silent - Silent playback infrastructure; Playback - Simulated screwpump noise.) Lines represent the model predictions; shaded area represent the 90% confidence intervals; an asterisk indicates a significant interaction between distance and treatment (*p* < 0.1)

Males of both sparrow species, by comparison, had elevated corticosterone levels in the presence of disturbances. Male Savannah Sparrows had higher stress responses when close to pumpjacks (*β* = −10.6, SE = 6.16, *p* = 0.09; Fig. 4a) and higher basal corticosterone near silent playback infrastructure (*β* = −0.68, SE = 0.34, *p* = 0.047). At the landscape scale, male Baird’s Sparrows had higher stress responses close to roads (*β* = −13.6, SE = 4.81, *p* = 0.01; Fig. 4b). Corticosterone levels of male Chestnut-collared Longspurs were independent of infrastructure and noise (*p* > 0.39). Other relationships were not significant.

## Discussion

Some grassland songbirds may avoid energy infrastructure, or experience reduced demographic success and provide less parental care near oil wells and roads (Bernath-Plaisted and Koper 2016; Nenninger and Koper 2018; Ng et al. 2019). This suggests that oil infrastructure may alter and reduce habitat quality for these species. Here, we explore two mechanisms that may help to explain these negative effects by demonstrating the potential for physiological impacts and changes in demographic distributions in grassland songbirds breeding near oil infrastructure. Further, we help to disentangle the impacts of physical infrastructure from associated anthropogenic noise by showing that noise per se does not appear to drive these effects.

Oil infrastructure, roads, or noise were correlated with altered corticosterone levels for each of our study species. Interpreting impacts of development on corticosterone levels is complex, as the relationships between corticosterone and stress are nonlinear; as the intensity of stress increases, corticosterone levels rise until the individual can no longer maintain increased expression, at which point corticosterone decreases as that individual is no longer able to cope with disturbance (Busch and Hayward 2009). As a result, either negative or positive relationships between disturbance and corticosterone levels can indicate physiological stress (Injaian et al. 2020). Chronic noise from energy development increased fecal corticosterone levels in Greater Sage-Grouse (*Centrocercus urophasianus*) on leks (Blickley et al. 2012), whereas female Tree Swallows (*Tachycineta bicolor*) showed reduced stress responses when exposed to chronic traffic noise (Injaian et al. 2018b). Our results, such as a trend for male sparrows to have an increased stress response near anthropogenic disturbances, suggest that some individuals may experience physiological stress when exposed to oil development and associated roads, which in turn may result in short- or long-term health effects (Busch and Hayward 2009).

We found that Savannah Sparrows and Baird’s Sparrows both avoided habitats that had demographic or physiological costs. For example, we found that Savannah Sparrows that occurred near oil wells, where nesting success is lower (Bernath-Plaisted and Koper 2016) and brood parasitism is higher (Bernath-Plaisted et al. 2017), were more likely to be low-quality individuals. This suggests that competitive sparrows preferentially selected habitats farther from wells, leaving the poor-quality habitat available for low-quality individuals. Similarly, Baird’s Sparrows exist at lower densities near roads (Nenninger and Koper 2018), a habitat where we found their stress response to be higher. In contrast, our results suggest that Chestnut-collared Longspurs, which are experiencing the steepest population declines of our focal species (Sauer et al. 2017), preferentially settled near infrastructure, since females captured there tended to be older and heavier. We speculate that Chestnut-collared Longspurs may be less sensitive to anthropogenically modified landscapes or are more limited in the space they are willing to occupy due to their specific habitat requirements. Cattle selectively graze near wells (Koper et al. 2014), which may create attractive habitat for short-grass specialists such as longspurs (Davis 2005; Lloyd and Martin 2005). However, if nesting near infrastructure increases physiological stress or decreases productivity, this mismatch in perceived and realized habitat quality may function as an ecological trap (Robertson and Hutto 2006). In contrast, Savannah Sparrows, a generalist that adapt readily to anthropogenic environments (Wheelright and Rising 2008), clearly use the presence of infrastructure and noise when making occupancy decisions. This further highlights the importance of taking a nuanced approach to managing a group of species that respond uniquely to energy development and habitat in general.

Males and females showed numerous differences in habitat selection, physiological responses to stressors, and in their relationship between morphology and productivity. For example, although male Chestnut-collared Longspurs showed no responses to wells or roads, we observed numerous significant effects of physical infrastructure and noise on female longspurs. While these results could have been influenced by our sampling of all females during the nestling stage, but not all males, there are also several biological factors that could explain these differences. Female longspurs appear to select the actual nest location within the territory of the pair, and are the primary nest builders and incubators (Lloyd and Martin 2005; Kirkham and Davis 2013). While male longspurs play an essential role in provisioning the female on the nest (Lynn and Wingfield 2003), they may be less vulnerable to disturbances than females as they can move away from the disturbance source in between feeding visits. Females may also be more susceptible to disturbances than males since they are more physiologically taxed during the breeding season from the investment of laying eggs, spending more time thermoregulating the nest, and thus less time foraging freely. These sex based differences in behavior, and inherent differences in current reproductive investment and future reproductive potential, affect the relative costs and benefits of reproduction in risky situations (Bókony et al. 2009). This could increase the overall cost of reproduction near oil infrastructure by creating conflicts in optimal reproductive strategies for each member of the parental pair.

Simulated oil well noise had few impacts on demographic distributions, basal corticosterone, or stress responses, and when we observed apparent effects of simulated oil well noise, they differed from effects of real wells. These findings contradict many previous studies that have concluded that noise influences productivity, stress, and settlement patterns in birds (Habib et al. 2007; Bayne et al. 2008; Francis et al. 2012; Kleist et al. 2016, 2018; Injaian et al. 2018a). In some of these studies, this discrepancy may result from a conflation of physical infrastructure and associated noise effects. Nonetheless, in situ manipulative noise experiments have demonstrated ecological effects of noise per se on birds (Blickley et al. 2012; Ware et al. 2015; Kleist et al. 2018). We suggest that birds might be able to adapt to oil well operating noise specifically because it is predictable and continuous (Blickley et al. 2012; Francis and Barber 2013; Curry et al. 2018). This is of key relevance to conservation and management because if noise is not the mechanism driving most of the ecological impacts observed in this system, then there would be little value in its mitigation. Similarly, Bernath-Plaisted and Koper (2016) and Nenninger and Koper (2018) both found that inactive oil wells had similar ecological effects to active oil wells, suggesting that neither noise nor human activity around wells explains their observed ecological effects. However, it is important to note that in some cases, there may be small additive effects of noise as well. We observed higher basal corticosterone of female Chestnut-collared Longspurs in proximity to simulated oil well noise, and longspurs have also been observed to decrease parental care of nests in noisy compared with quiet sites (Ng et al. 2019). This is important from a management perspective, as the negative impacts of anthropogenic noise could facilitate further declines of this species at risk. Additionally, the idiosyncratic response of many species to infrastructure and noise further emphasizes the importance of including species of management concern in studies such as ours.

A recent study on impacts of noise on three cavity-nesting bird species found that basal corticosterone was consistently lower near chronic anthropogenic noise from natural gas compressor stations and attributed the associated declines in productivity to that increased noise (Kleist et al. 2018). This differs from our results, suggesting a more pervasive impact of noise than we detected. Natural gas compressor stations are much louder than oil wells (Rosa et al. 2015; Kleist et al. 2018), so perhaps this disturbance is enough to push the surrounding individuals past the tipping point of maximum corticosterone output. Cavity-nesting birds are also distinct in both taxonomy and life history from the families represented in our study (Passerellidae, Calcariidae), so it is likely that their capacity to respond to disturbances is bounded by different thresholds (Busch and Hayward 2009). Finally, it is also possible that because grasslands are filled with an abundance of natural noises (e.g. wind, birdsong, insects) with which prairie songbirds have evolved, our focal species are well adapted to noisy environments and can compensate for some types of anthropogenic noise (Curry et al. 2017, 2018).

Although physical infrastructure seems to be a stronger driver of ecological effects than noise, these effects are not consistent among structure types. Both screwpumps and pumpjacks fulfill a similar function in bringing oil to the surface, they have substantially different ecological impacts. For example, female Chestnut-collared Longspurs had higher basal corticosterone near simulated screwpump noise, but lower basal corticosterone near pumpjacks. Impacts of wells on abundance (Nenninger and Koper 2018), nesting success (Bernath-Plaisted and Koper 2016), vocalizations (Warrington et al. 2018), and behavioral adaptations (Curry et al. 2018) also vary with infrastructure type, presumably as a result of moderate differences in above-ground profile, height, and movement, as well as acoustic characteristics of the sounds they emit (Warrington et al. 2018). However, neither type of pump is consistently more problematic – for example, screwpumps have a greater impact on nesting success (Bernath-Plaisted and Koper 2016), whereas our present study demonstrated that pumpjacks have a greater impact on Savannah Sparrow stress responses. Consequently, neither well type can be consistently recommended as having a smaller ecological footprint, making it difficult to translate these differences into management solutions.

## Conclusions

The direct habitat loss and associated disturbances from oil development in the Northern Great Plain of North America pose a significant threat to birds that rely on the remaining intact patches of grassland habitat, especially in Canada, where habitat loss has been particularly severe (Gage et al. 2016; World Wildlife Fund 2021). Luckily, across several grassland species, noise associated with oil wells appears to be relatively unimportant in comparison with other effects associated with the presence of physical infrastructure. For those species, this would mean the size of the disturbance footprint may not be as large or pervasive as for noise-sensitive species (Francis and Barber 2013). However, because corticosterone levels of female Chestnut-collared Longspurs varied with noise per se, minimizing extent of both physical infrastructure and noise would be beneficial. We demonstrated that different species show different responses to oil development, so a diversity of mitigation strategies is required to mediate the risks to wildlife posed by this industry. Clustering above-ground well heads onto existing well pads and accessing oil reserves using directional drilling (Thompson et al. 2015), decommissioning old well heads, and reclaiming rarely used roads would all contribute to reducing disturbances from the built environment, while mufflers can decrease amplitude of operating noise of wells. Ultimately, however, the best mitigation for the effects of oil development would be to reduce our dependence on fossil fuels, thereby limiting further environmental damage from oil extraction and human induced climate change.

## Supplementary information

Supplementary Information
