# Supplementary material for: Oil Infrastructure has Greater Impact than Noise on Stress and Habitat Selection in Three Grassland Songbirds
Source: Environ Manage. 2022 Dec 2;71(2):393–404. doi: 10.1007/s00267-022-01752-2 (PMC9892115; doi:10.1007/s00267-022-01752-2)
Supplement: Supplementary file 1 — Supplementary Information [file 267_2022_1752_MOESM1_ESM.docx]

Supplemental Information

Study Area

All research occurred within 60 km of Brooks, Alberta, Canada (50°33′51″N 111°53′56″W, 760 MASL) on land owned by the Eastern Irrigation District (EID), a private landowner that primarily uses grasslands for grazing cattle and owns more than 282,800 ha in Alberta. These lands are annually grazed mixed-grass prairie, consisting mainly of native grasses and forbs: needle and thread (*Hesperostipa comata*), porcupine grass (*Hesperostipa spartea*), blue grama (*Bouteloua gracilis*), western wheatgrass (*Pascopyrum smithii*), pasture sagewort (*Artemisia frigida*), and silver sagebrush (*Artemisia cana*), intermixed with a low abundance of invasive grasses and forbs, including goatsbeard (*Tragopogon dubius*), dandelion (*Taraxacum officinale*), crested wheatgrass (*Agropyron cristatum*), and smooth brome (*Bromus inermis*). Brooks receives an average of 252.6 mm of rainfall annually (347.5 mm total precipitation; Environment Canada 2018) and is on the edge of the prairie pothole region. Pastures are divided by three-wire fences into Sections (1.6 x 1.6 km) or larger, which are also often accompanied by gravel, or occasionally paved, range roads (1.6 by 1.6 to 3.2-km grid). Active lease sites are operated for oil and natural gas on EID lands around Brooks. Lease sites are 1 hectare (100 m x 100 m) and can have up to 3 well heads per lease. Oil infrastructure generally consists of an oil well pad (usually approximately 50 m x 50 m of level gravel), which is connected to the rural road system by a gravel or dirt service road, and one or more centralized oil wells (~ 2m - 4m high) that generate noise and have pump components that sweep up and down vertically or spin horizontally.

Corticosterone quantification

To determine pre-capture circulating corticosterone levels (basal corticosterone), 70 μL of blood was taken from the brachial vein within three minutes of capture (Romero and Reed, 2005). Individuals were then subjected to a standardized 12-minute stress handling protocol (Wingfield et al., 1992), during which time we banded and measured birds, followed by an additional 30-70 μL blood sample to determine the increase in corticosterone in response to handling (stress response).To maintain consistency in the protocol among species, we used the same length of stress handling protocol for all three species. Peak increase in corticosterone for Chestnut-collared Longspurs occurs after approximately 10 minutes of handling (Lynn et al., 2003). We therefore choose a stress handling protocol of 12 minutes to maximize the observed increase in corticosterone while minimizing total handling time and therefore the potential for adverse effects from capture. Blood samples were collected via brachial venipuncture using a heparinized micro-capillary tube and kept on ice (< 6 hours) until the plasma was separated by centrifuge (10 minutes at 15,000 G) and then frozen at -20ºC until extraction.

Plasma corticosterone concentrations were determined using a radioimmunoassay (Crossin et al., 2012; Sheriff et al., 2011; Wingfield et al., 1992) from the blood plasma. Samples were extracted using absolute ethanol in a 12:1 ratio with the plasma volume, centrifuged at 6000 G for 3 minutes, and the supernatant decanted. This process was repeated, and the supernatants were pooled. This extract was then dried and frozen until assayed. Extraction efficiency of corticosterone from plasma samples was 113.2%. On the day of the assay procedure, samples were allowed to reconstitute in RIA buffer (phosphate buffer, NaCl, and Bovine serum albumin), to an appropriate dilution such that 100 μL of buffer constituted 4-20 μL of plasma depending on the species and sample, for one hour after an initial agitation of 5 seconds in an orbital shaker (vortex). Each assay tube contained 100 μL each of 1:6000 diluted corticosterone antibody (Antibodies-online, cat no. ABIN343319), ^3^H-labeled corticosterone, and reconstituted sample or corticosterone standard. After incubation, unbound corticosterone was removed using an activated charcoal separation buffer (RIA buffer, charcoal, dextran), the supernatant was decanted into scintillation vials, and 4mL of scintillation fluid (UltimaGold; PerkinElmer) was added. Vials were counted in a scintillation counter for 5 minutes each. Sample corticosterone values were interpolated from the dose-sigmoidal response curve produced by a serial dilution of corticosterone standards included in each assay using Prism 6 (Graphpad). Inter-assay variability was 14.5%, intra-assay variability was 13.4%, and parallelism was achieved.

The assay-specific upper and lower detection limits were 3.0 ng/mL and 0.2 ng/mL of corticosterone respectively. Some samples failed to produce interpretable corticosterone values due to low concentrations of corticosterone in the plasma sample and could not be assigned specific concentrations of corticosterone. Since the concentration of corticosterone is calculated post-hoc based on the absolute amounts of corticosterone and the volume of plasma represented per assay tube, it is possible to calculate the minimum concentration of corticosterone necessary in a sample of a given volume to produce an interpretable value on the dose-sigmoidal response curve. If samples had large enough plasma volumes to detect corticosterone above 1-2 ng/mL in the sample, then they were assigned the assay specific minimum detectable corticosterone value based on the sample’s plasma volume and were included in subsequent analyses. Samples that produced interpretable values above the detectable limit of the assay were similarly assigned the assay specific maximum detectable corticosterone value and were included in subsequent analyses (e.g., see Kleist et al., 2018). While we cannot be certain of the concentration of corticosterone in the aforementioned samples, this method of assigning corticosterone values is conservative when assigning low volume samples that biologically must fall below 0 and the minimum detectable value (1-2 ng/mL).

Literature Cited

Crossin, G., Trathan, P.N., Phillips, R. a., Gorman, K.B., Dawson, A., Sakamoto, K.Q., Williams, T.D., 2012. Corticosterone predicts foraging behavior and parental care in Macaroni Penguins. Am. Nat. 180, E31–E41. https://doi.org/10.1086/666001

Kleist, N.J., Guralnick, R.P., Cruz, A., Lowry, C.A., Francis, C.D., 2018. Chronic anthropogenic noise disrupts glucocorticoid signaling and has multiple effects on fitness in an avian community. Proc. Natl. Acad. Sci. 115, E648–E657. https://doi.org/10.1073/pnas.1709200115

Lynn, S., Hunt, K., Wingfield, J.C., 2003. Ecological factors affecting the adrenocortical response to stress in chestnut‐collared and McCown’s longspurs (*Calcarius ornatus*, *Calcarius mccownii*). Physiol. Biochem. Zool. 76, 566–576.

Romero, L.M., Reed, J., 2005. Collecting baseline corticosterone samples in the field: is under 3 min good enough? Comp. Biochem. Physiol. 140, 73–79. https://doi.org/10.1016/j.cbpb.2004.11.004

Rosa, P., Koper, N., 2018. Integrating multiple disciplines to understand effects of anthropogenic noise on animal communication: Ecosphere 9, e02127. https://doi.org/10.1002/ecs2.2127

Rosa, P., Swider, C.R., Leston, L., Koper, N., 2015. Disentangling effects of noise from presence of anthropogenic infrastructure: Design and testing of system for large-scale playback experiments. Wildl. Soc. Bull. 39, 364–372. https://doi.org/10.1002/wsb.546

Sheriff, M.J., Dantzer, B., Delehanty, B., Palme, R., Boonstra, R., 2011. Measuring stress in wildlife: Techniques for quantifying glucocorticoids. Oecologia 869–887. https://doi.org/10.1007/s00442-011-1943-y

Wingfield, J.C., Vleck, C.M., Moore, M.C., 1992. Seasonal changes of the adrenocortical response to stress in birds of the Sonoran desert. J. Exp. Zool. 264, 419–428. https://doi.org/10.1002/jez.1402640407


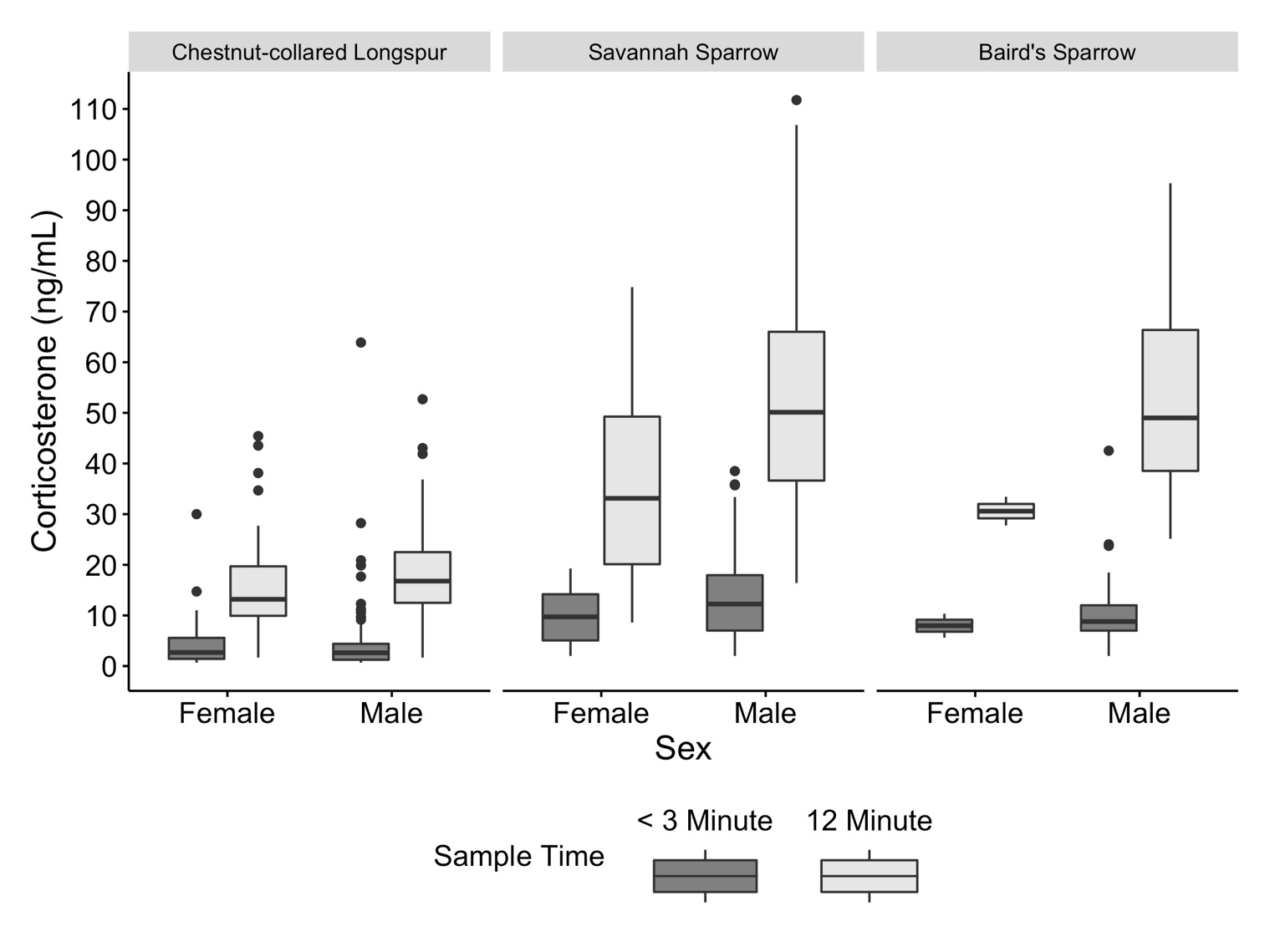


Figure S1.Corticosterone values from the basal (<3min; blue) and stress response (12min; red) sample from southern Alberta, 2015 – 2016 showing species and sex needing separate analysis.

*Table S1. Influence of female weight, age (see footnote), size (tarsus) on total nest biomass(combined weight off all nestlings) and individual nestling weight. Additionally, influence of brood size and average nestling age on total nestling biomass, and influence of nestling age on individual nestling mass.*

|  | CCLO Nest Biomass | | | SAVS Nest Biomass | | | CCLO Nestling Weight | | | SAVS Nestling Weight | | |
| --- | --- | --- | --- | --- | --- | --- | --- | --- | --- | --- | --- | --- |
| *Predictor* | *Beta* | *SE* | *p* | *Beta* | *SE* | *p* | *Beta* | *SE* | *p* | *Beta* | *SE* | *p* |
| (Intercept) | 20.7 | 54.0 | 0.70 | -121 | 466 | 0.80 | 4.56 | 8.36 | 0.58 | -6.08 | 33.9 | 0.86 |
| Female Weight | 3.29 | 1.46 | **0.03** | 5.05 | 5.78 | 0.41 | 0.14 | 0.22 | 0.51 | 0.97 | 0.42 | **0.05** |
| Female Age: SY | -2.51 | 3.73 | 0.50 | -0.49 | 19.0 | 0.98 | -0.33 | 0.57 | 0.56 | -2.24 | 1.34 | 0.14 |
| Female Tarsus | -4.36 | 2.67 | 0.11 | 2.67 | 20.2 | 0.90 | 0.03 | 0.42 | 0.94 | -0.58 | 1.49 | 0.71 |
| Brood Size | 12.1 | 1.36 | **<0.01** | 4.88 | 7.91 | 0.56 |  |  |  |  |  |  |
| Average Nestling Age | 0.50 | 2.27 | 0.82 | 1.13 | 14.2 | 0.94 |  |  |  |  |  |  |
| Nestling age |  |  |  |  |  |  | 0.78 | 0.34 | **0.02** | 2.13 | 1.04 | **0.08** |
| **Random Effects** | | | | | | | | | | | | |
| σ^2^ |  | | |  | | | 3.14 | | | 1.21 | | |
| τ_00_ |  | | |  | | | 1.88 _nest_id_ | | | 1.44 _nest_id_ | | |
| ICC |  | | |  | | | 0.37 _nest_id_ | | | 0.54 _nest_id_ | | |
| Observations | 40 | | | 12 | | | 178 | | | 36 | | |
| R^2^ / adjusted R^2^ | 0.738 / 0.700 | | | 0.159 / -0.541 | | | 0.067 / 0.417 | | | 0.338 / 0.699 | | |

*SY: Second year bird – a bird in its second calendar year, one year after hatching.*

*Table S2. Influence of distance from oil well and roads on age class (Second Year or After Second Year) of female and male Chestnut-collared Longspur, male Baird’s Sparrow, and male and female Savannah Sparrow. Birds were captured during the breeding seasons of 2015 to 2017 in Southern Alberta. Distances are from the nearest infrastructure and road measured in Log(m). Model was fitted with a binomial distribution.*

|  | Female Chestnut-collared Longspur | | | Male Chestnut-collared Longspur | | | Male Baird’s Sparrow | | | Female Savannah Sparrow | | | Male Savannah Sparrow | | |
| --- | --- | --- | --- | --- | --- | --- | --- | --- | --- | --- | --- | --- | --- | --- | --- |
| *Predictor* | *Beta* | *SE* | *p* | *Beta* | *SE* | *p* | *Beta* | *SE* | *p* | *Beta* | *SE* | *p* | *Beta* | *SE* | *p* |
| (Intercept) | 2.81 | 1.61 | **0.08** | 1.94 | 1.47 | 0.18 | 0.67 | 2.83 | 0.81 | -2.70 | 3.78 | 0.47 | -2.46 | 1.37 | **0.07** |
| Distance from Oil Well | -0.53 | 0.28 | **0.06** | -0.11 | 0.25 | 0.66 | -0.07 | 0.38 | 0.84 | -0.42 | 0.53 | 0.42 | 0.32 | 0.22 | 0.14 |
| Distance from Road | 0.20 | 0.25 | 0.42 | 0.01 | 0.20 | 0.95 | -0.01 | 0.40 | 0.98 | 0.90 | 0.70 | 0.20 | 0.07 | 0.28 | 0.80 |
| n | 73 | | | 102 | | | 74 | | | 23 | | | 105 | | |
| Cox & Snell's R^2^ / Nagelkerke's R^2^ | 0.050 / 0.069 | | | 0.002 / 0.003 | | | 0.001 / 0.001 | | | 0.082 / 0.110 | | | 0.039 / 0.051 | | |

*Table S3. Influence of distance from oil well and roads on size (tarsus length in mm) of female and male Chestnut-collared Longspur, male Baird’s Sparrow, and male and female Savannah Sparrow. Birds were captured during the breeding seasons of 2015 to 2017 in Southern Alberta. Distances are from the nearest infrastructure and road measured in Log(m).*

|  | Female Chestnut-collared Longspur | | | Male Chestnut-collared Longspur | | | Male Baird’s Sparrow | | | Female Savannah Sparrow | | | Male Savannah Sparrow | | |  |
| --- | --- | --- | --- | --- | --- | --- | --- | --- | --- | --- | --- | --- | --- | --- | --- | --- |
| *Predictor* | *Beta* | *SE* | *p* | *Beta* | *SE* | *p* | *Beta* | *SE* | *p* | *Beta* | *SE* | *p* | *Beta* | *SE* | *p* | |
| Intercept | 19.2 | 0.41 | **<0.01** | 19.7 | 0.42 | **<0.01** | 20.1 | 0.97 | **<0.01** | 19.3 | 1.28 | **<0.01** | 20.8 | 0.5 | **<0.01** | |
| Distance from Oil Well | -0.05 | 0.07 | 0.48 | -0.05 | 0.07 | 0.50 | 0.03 | 0.13 | 0.82 | 0.01 | 0.18 | 0.96 | -0.05 | 0.08 | 0.47 | |
| Distance from Road | 0.15 | 0.07 | **0.03** | 0.07 | 0.06 | 0.24 | 0.19 | 0.14 | 0.16 | 0.06 | 0.21 | 0.77 | -0.02 | 0.10 | 0.82 | |
| n | 73 | | | 102 | | | 74 | | | 23 | | | 105 | | |  |
| R^2^/  adjusted R^2^ | 0.068 / 0.042 | | | 0.014 / -0.006 | | | 0.035 / 0.008 | | | 0.006 / -0.093 | | | 0.012 / -0.008 | | |  |

*Table S4. Influence of distance from oil well and roads on weight (g) of female and male Chestnut-collared Longspur, male Baird’s Sparrow, and male and female Savannah Sparrow. Birds were captured during the breeding seasons of 2015 to 2017 in Southern Alberta. Distances are from the nearest infrastructure and road measured in Log(m).*

|  | Female Chestnut-collared Longspur | | | Male Chestnut-collared Longspur | | | Male Baird’s Sparrow | | | Female Savannah Sparrow | | | Male Savannah Sparrow | | |
| --- | --- | --- | --- | --- | --- | --- | --- | --- | --- | --- | --- | --- | --- | --- | --- |
| *Predictor* | *Beta* | *SE* | *p* | *Beta* | *SE* | *p* | *Beta* | *SE* | *p* | *Beta* | *SE* | *p* | *Beta* | *SE* | *p* |
| (Intercept) | 23.8 | 1.49 | **<0.01** | 20.80 | 1.12 | **<0.01** | 23.8 | 1.64 | **<0.01** | 18.3 | 3.32 | **<0.01** | 18.9 | 1.06 | **<0.01** |
| Distance from Oil Well | -0.26 | 0.14 | **0.06** | -0.08 | 0.10 | 0.40 | -0.13 | 0.16 | 0.43 | -0.03 | 0.27 | 0.91 | 0.07 | 0.11 | 0.54 |
| Distance from Road | -0.02 | 0.13 | 0.90 | 0.03 | 0.08 | 0.68 | -0.16 | 0.17 | 0.35 | 0.04 | 0.32 | 0.89 | -0.08 | 0.14 | 0.59 |
| Season Day | -0.02 | 0.01 | **0.02** | -0.01 | 0.01 | 0.32 | -0.02 | 0.01 | **0.01** | -0.01 | 0.01 | 0.35 | -0.01 | 0.01 | 0.29 |
| n | 72 | | | 101 | | | 73 | | | 23 | | | 103 | | |
| R^2^ / adjusted R^2^ | 0.135 / 0.096 | | | 0.018 / -0.012 | | | 0.131 / 0.093 | | | 0.051 / -0.099 | | | 0.019 / -0.010 | | |

*Table S5. Influence of distance to type of oil infrastructure or simulated screwpump noise on age class (second year or After Second Year) of female and male Chestnut-collared Longspur, male Baird’s Sparrow, and male and female Savannah Sparrow. Birds were captured during the breeding seasons of 2015 to 2017 in Southern Alberta. Distances from center of each site or infrastructure/ playback unit and are measured in Log(m). Model was fitted with a binomial distribution.*

|  | Female Chestnut-collared Longspur | | | Male Chestnut-collared Longspur | | | Male Baird’s Sparrow | | | Female Savannah Sparrow | | | Male Savannah Sparrow | | |
| --- | --- | --- | --- | --- | --- | --- | --- | --- | --- | --- | --- | --- | --- | --- | --- |
| *Predictors* | *Beta* | *SE* | *p* | *Beta* | *SE* | *p* | *Beta* | *SE* | *p* | *Beta* | *SE* | *p* | *Beta* | *SE* | *p* |
| (Intercept) | 5.35 | 3.81 | 0.160 | 0.37 | 3.34 | 0.912 | 0.27 | 2.86 | 0.926 | 2.84 | 6.46 | 0.661 | 0.61 | 2.57 | 0.814 |
| Distance to Control | -0.91 | 0.68 | 0.180 | 0.15 | 0.60 | 0.807 | -0.08 | 0.49 | 0.872 | -0.58 | 1.14 | 0.608 | -0.09 | 0.46 | 0.839 |
| Distance to Silent | 0.57 | 1.32 | 0.666 | -1.71 | 1.45 | 0.240 | 2.20 | 1.96 | 0.261 |  |  |  | 0.26 | 1.04 | 0.804 |
| Distance to Simulated screwpump | 1.00 | 0.94 | 0.286 | 0.73 | 1.00 | 0.463 | -2.14 | 2.58 | 0.408 | -7.43 | 13.25 | 0.575 | -1.51 | 2.94 | 0.608 |
| Distance to Screwpump | 0.89 | 1.04 | 0.390 | 0.52 | 0.83 | 0.532 | 0.58 | 1.18 | 0.626 | -0.29 | 2.26 | 0.899 | 0.26 | 0.77 | 0.732 |
| Distance to Pumpjack | -3.98 | 3.42 | 0.245 | -2.84 | 1.91 | 0.137 |  |  |  |  |  |  | 0.49 | 0.86 | 0.566 |
| Observations | 95 | | | 134 | | | 78 | | | 25 | | | 121 | | |
| Cox & Snell's R^2^ / Nagelkerke's R^2^ | 0.090 / 0.125 | | | 0.062 / 0.096 | | | 0.069 / 0.092 | | | 0.159 / 0.213 | | | 0.074 / 0.099 | | |

‘*Pumpjack’ refers to propane-powered pumpjack oil wells that draw oil from underground using a vertical see-saw motion. ‘Screwpump’ refers to propane-powered screwpump oil wells, another type of oil well without the associated large movements of a ‘pumpjack. ‘Simulated screwpump’ is a high-fidelity experiential noise treatment, broadcasting the sound of a screwpump oil well 24h a day in field absent of oil infrastructure throughout the breeding season. ‘Silent’ treatments have the same fencing and build as the playback infrastructure, but do not broadcast noise. Controls are field with no infrastructure nor experimental playback manipulations.*

*Table S6. Influence of distance to type of oil infrastructure or simulated screwpump noise on size (Tarsus length mm) of female and male Chestnut-collared Longspur, male Baird’s Sparrow, and male and female Savannah Sparrow. Birds were captured during the breeding seasons of 2015 to 2017 in Southern Alberta. Distances from center of each site or infrastructure/ playback unit and are measured in Log(m).*

|  | Female Chestnut-collared Longspur | | | Male Chestnut-collared Longspur | | | Male Baird’s Sparrow | | | Female Savannah Sparrow | | | Male Savannah Sparrow | | |
| --- | --- | --- | --- | --- | --- | --- | --- | --- | --- | --- | --- | --- | --- | --- | --- |
| *Predictors* | *Beta* | *SE* | *p* | *Beta* | *SE* | *p* | *Beta* | *SE* | *p* | *Beta* | *SE* | *p* | *Beta* | *SE* | *p* |
| (Intercept) | 19.66 | 0.88 | **<0.001** | 17.85 | 1.07 | **<0.001** | 20.49 | 0.98 | **<0.001** | 22.62 | 2.12 | **<0.001** | 21.17 | 0.86 | **<0.001** |
| Distance to Control | -0.00 | 0.16 | 0.995 | 0.35 | 0.19 | **0.074** | 0.18 | 0.17 | 0.288 | -0.49 | 0.37 | 0.206 | -0.16 | 0.15 | 0.300 |
| Distance to Silent | 0.36 | 0.31 | 0.238 | -0.50 | 0.33 | 0.129 | -0.47 | 0.52 | 0.366 |  |  |  | 0.24 | 0.33 | 0.477 |
| Distance to Simulated screwpump | -0.10 | 0.24 | 0.669 | -0.49 | 0.31 | 0.121 | -0.38 | 0.58 | 0.519 | -0.72 | 1.36 | 0.601 | -0.26 | 0.70 | 0.705 |
| Distance to Screwpump | 0.04 | 0.27 | 0.884 | -0.27 | 0.26 | 0.304 | -0.82 | 0.40 | **0.044** | 1.41 | 0.74 | **0.074** | 0.20 | 0.25 | 0.425 |
| Distance to Pumpjack | -0.11 | 0.40 | 0.789 | -0.45 | 0.41 | 0.273 |  |  |  |  |  |  | -0.27 | 0.29 | 0.350 |
| Observations | 95 | | | 134 | | | 78 | | | 25 | | | 121 | | |
| R^2^ / adjusted R^2^ | 0.081 / -0.016 | | | 0.070 / 0.002 | | | 0.090 / -0.001 | | | 0.268 / 0.023 | | | 0.080 / 0.005 | | |

‘*Pumpjack’ refers to propane-powered pumpjack oil wells that draw oil from underground using a vertical see-saw motion. ‘Screwpump’ refers to propane-powered screwpump oil wells, another type of oil well without the associated large movements of a pumpjack. ‘Playback’ is a high-fidelity experiential noise treatment, broadcasting the sound of a screwpump oil well 24h a day in field absent of oil infrastructure throughout the breeding season. ‘Silent’ treatments have the same fencing and build as the playback infrastructure, but do not broadcast noise. Controls are field with no infrastructure nor experimental playback manipulations.*

*Table S7. Influence of distance to type of oil infrastructure or simulated screwpump noise on weight (g) of female and male Chestnut-collared Longspur, male Baird’s Sparrow, and male and female Savannah Sparrow. Birds were captured during the breeding seasons of 2015 to 2017 in Southern Alberta. Distances from center of each site or infrastructure/ playback unit and are measured in Log(m).*

|  | Female Chestnut-collared Longspur | | | Male Chestnut-collared Longspur | | | Male Baird’s Sparrow | | | Female Savannah Sparrow | | | Male Savannah Sparrow | | |
| --- | --- | --- | --- | --- | --- | --- | --- | --- | --- | --- | --- | --- | --- | --- | --- |
| *Predictors* | *Beta* | *SE* | *p* | *Beta* | *SE* | *p* | *Beta* | *SE* | *p* | *Beta* | *SE* | *p* | *Beta* | *SE* | *p* |
| (Intercept) | 22.44 | 1.93 | **<0.001** | 20.33 | 1.84 | **<0.001** | 20.36 | 1.72 | **<0.001** | 21.21 | 3.41 | **<0.001** | 21.42 | 1.51 | **<0.001** |
| Distance to Control | -0.17 | 0.30 | 0.572 | 0.19 | 0.30 | 0.522 | 0.12 | 0.23 | 0.601 | -0.70 | 0.61 | 0.269 | -0.42 | 0.23 | **0.063** |
| Distance to Silent | -0.01 | 0.01 | **0.032** | -0.01 | 0.01 | **0.068** | -0.01 | 0.01 | **0.045** | -0.00 | 0.01 | 0.820 | -0.01 | 0.01 | 0.189 |
| Distance to Simulated screwpump | -0.48 | 0.58 | 0.417 | -0.75 | 0.51 | 0.142 | -0.54 | 0.70 | 0.449 |  |  |  | 0.89 | 0.49 | **0.073** |
| Distance to Screwpump | 0.10 | 0.45 | 0.833 | -0.52 | 0.49 | 0.287 | -0.73 | 0.81 | 0.373 | -1.87 | 2.15 | 0.397 | -0.40 | 1.03 | 0.700 |
| Distance to Pumpjack | -0.72 | 0.50 | 0.154 | 0.17 | 0.40 | 0.681 | -0.82 | 0.55 | 0.137 | 0.88 | 1.15 | 0.453 | 0.83 | 0.37 | **0.028** |
| Season Day | -0.74 | 0.74 | 0.320 | -0.45 | 0.64 | 0.482 |  |  |  |  |  |  | -0.03 | 0.42 | 0.938 |
| Observations | 94 | | | 133 | | | 77 | | | 25 | | | 119 | | |
| R^2^ / adjusted R^2^ | 0.163 / 0.062 | | | 0.110 / 0.037 | | | 0.138 / 0.036 | | | 0.356 / 0.091 | | | 0.113 / 0.031 | | |

‘*Pumpjack’ refers to propane-powered pumpjack oil wells that draw oil from underground using a vertical see-saw motion. ‘Screwpump’ refers to propane-powered screwpump oil wells, another type of oil well without the associated large movements of a pumpjack. ‘Simulated screwpump’ is a high-fidelity experiential noise treatment, broadcasting the sound of a screwpump oil well 24h a day in field absent of oil infrastructure throughout the breeding season. ‘Silent’ treatments have the same fencing and build as the playback infrastructure, but do not broadcast noise. Controls are field with no infrastructure nor experimental playback manipulations.*

*Table S8. Influence of weight, age (see footer), season, and time of day on basal corticosterone levels (Log (ng/ml)) in female and male Chestnut-collared Longspur, male Baird’s Sparrow, and male and female Savannah Sparrow. Samples were obtained during the breeding seasons of 2015 to 2016 in Southern Alberta.*

|  | Female Chestnut-collared Longspur | | | Male Chestnut-collared Longspur | | | Male Baird’s Sparrow | | | Female Savannah Sparrow | | | Male Savannah Sparrow | | | | |
| --- | --- | --- | --- | --- | --- | --- | --- | --- | --- | --- | --- | --- | --- | --- | --- | --- | --- |
| *Predictors* | *Beta* | *SE* | *p* | *Beta* | *SE* | *p* | *Beta* | *SE* | *p* | *Beta* | *SE* | *p* | *Beta* | *SE* | *p* | | |
| (Intercept) | 1.35 | 2.15 | 0.53 | 1.95 | 1.74 | 0.26 | 10.3 | 2.24 | **<0.01** | 8.79 | 5.95 | 0.16 | 6.64 | 1.56 | | **<0.01** |  |
| Weight | -0.07 | 0.09 | 0.41 | -0.02 | 0.07 | 0.80 | -0.28 | 0.10 | **0.01** | -0.32 | 0.27 | 0.26 | -0.10 | 0.07 | | 0.18 |  |
| Age: ASY | 0.08 | 0.21 | 0.71 | 0.35 | 0.21 | 0.09 | 0.28 | 0.17 | 0.12 | -0.05 | 0.39 | 0.89 | 0.31 | 0.14 | | **0.03** |  |
| Season Day | 0.01 | 0.01 | 0.11 | 0.00 | 0.00 | 0.97 | -0.01 | 0.01 | **0.01** | -0.01 | 0.02 | 0.47 | -0.02 | 0.00 | | **<0.01** |  |
| Time of Day | -0.00 | 0.00 | 0.42 | -0.00 | 0.00 | 0.06 | -0.00 | 0.00 | 0.20 | 0.00 | 0.00 | 0.75 | 0.00 | 0.00 | | 0.88 |  |
| n | 82 | | | 114 | | | 46 | | | 20 | | | 103 | | | | |
| R^2^/  adjusted R^2^ | 0.057 / 0.008 | | | 0.048 / 0.013 | | | 0.297 / 0.228 | | | 0.113 / -0.123 | | | 0.204 / 0.171 | | | | |

*ASY: After second year bird – a bird in its third calendar year since hatching, indicating a mature adult bird.*

*Table S9. Influence of basal corticosterone, weight, age (see footer), season, and time of day on stress response corticosterone levels (ng/ml) from a 12-minute stress handling protocol for female and male Chestnut-collared Longspur, male Baird’s Sparrow, and male and female Savannah Sparrow. Samples were obtained during the breeding seasons of 2015 to 2016 in Southern Alberta.*

|  | Female Chestnut-collared Longspur | | | Male Chestnut-collared Longspur | | | Male Baird’s Sparrow | | | Female Savannah Sparrow | | | Male Savannah Sparrow | | |
| --- | --- | --- | --- | --- | --- | --- | --- | --- | --- | --- | --- | --- | --- | --- | --- |
| *Predictor* | *Beta* | *SE* | *p* | *Beta* | *SE* | *p* | *Beta* | *SE* | *p* | *Beta* | *SE* | *p* | *Beta* | *SE* | *p* |
| (Intercept) | 33.2 | 21.4 | 0.12 | 32.4 | 15.4 | **0.04** | 151 | 81.0 | 0.07 | 28.6 | 103 | 0.79 | 82.8 | 43.4 | **0.06** |
| Basal CORT | -0.27 | 1.13 | 0.82 | 0.81 | 0.87 | 0.35 | -0.19 | 4.75 | 0.97 | 9.30 | 4.66 | **0.07** | 3.34 | 2.59 | 0.20 |
| Weight | -0.68 | 0.91 | 0.46 | -0.32 | 0.64 | 0.62 | -2.44 | 3.17 | 0.45 | -3.15 | 4.54 | 0.50 | -1.21 | 1.87 | 0.52 |
| Age: ASY | 0.41 | 2.02 | 0.84 | -2.89 | 1.86 | 0.12 | 7.76 | 5.30 | 0.15 | 12.7 | 6.53 | **0.07** | -3.53 | 3.61 | 0.33 |
| Season Day | 0.01 | 0.05 | 0.84 | -0.05 | 0.04 | 0.22 | -0.17 | 0.17 | 0.33 | 0.02 | 0.28 | 0.95 | -0.14 | 0.11 | 0.21 |
| Time of Day | -0.02 | 0.01 | **0.03** | -0.01 | 0.01 | 0.61 | -0.09 | 0.03 | **0.01** | 0.04 | 0.05 | 0.37 | -0.01 | 0.02 | 0.49 |
| n | 79 | | | 110 | | | 45 | | | 19 | | | 101 | | |
| R^2^/  adjusted R^2^ | 0.071 / 0.007 | | | 0.054 / 0.008 | | | 0.239 / 0.141 | | | 0.538 / 0.360 | | | 0.071 / 0.022 | | |

*ASY: After second year bird – a bird in its third calendar year since hatching, indicating a mature adult bird.*

*Table S10. Influence of distance from any type of oil well (Log(m)), distance from road (Log(m)), weight, age (see footer), and day on basal corticosterone levels (Log (ng/ml)) in female and male Chestnut-collared Longspur, male Baird’s Sparrow, and male and female Savannah Sparrow. Samples were obtained during the breeding seasons of 2015 to 2016 in Southern Alberta.*

|  | Female Chestnut-collared Longspur | | | Male Chestnut-collared Longspur | | | Male Baird’s Sparrow | | | Female Savannah Sparrow | | | Male Savannah Sparrow | | |
| --- | --- | --- | --- | --- | --- | --- | --- | --- | --- | --- | --- | --- | --- | --- | --- |
| *Predictor* | *Beta* | *SE* | *p* | *Beta* | *SE* | *p* | *Beta* | *SE* | *p* | *Beta* | *SE* | *p* | *Beta* | *SE* | *p* |
| (Intercept) | 1.40 | 0.56 | **0.02** | 0.76 | 0.56 | 0.18 | 12.4 | 3.39 | **0.01** | 1.00 | 1.49 | 0.52 | 4.72 | 0.66 | **<0.01** |
| Distance from Oil Well | 0.03 | 0.10 | 0.74 | 0.04 | 0.09 | 0.69 | -0.15 | 0.17 | 0.38 | -0.02 | 0.23 | 0.94 | -0.03 | 0.07 | 0.69 |
| Distance from Road | -0.11 | 0.09 | 0.22 | -0.05 | 0.07 | 0.50 | -0.16 | 0.19 | 0.40 | 0.19 | 0.26 | 0.48 | -0.01 | 0.10 | 0.91 |
| Age: ASY |  |  |  | 0.20 | 0.24 | 0.40 |  |  |  |  |  |  | 0.34 | 0.13 | **0.01** |
| Weight |  |  |  |  |  |  | -0.30 | 0.13 | **0.02** |  |  |  |  |  |  |
| Season Day |  |  |  |  |  |  | -0.01 | 0.01 | **0.01** |  |  |  | -0.01 | 0.00 | **<0.01** |
| n | 63 | | | 88 | | | 42 | | | 18 | | | 89 | | |
| R^2^ /  adjusted R^2^ | 0.026 / -0.006 | | | 0.013 / -0.022 | | | 0.224 / 0.140 | | | 0.041 / -0.087 | | | 0.210 / 0.173 | | |

*ASY: After second year bird – a bird in its third calendar year since hatching, indicating a mature adult bird.*

*Table S11. Influence of distance from any type of oil well (Log(m)), distance from road (Log(m)), time of day, and basal corticosterone level on stress response corticosterone levels (ng/ml) in female and male Chestnut-collared Longspur, male Baird’s Sparrow, and male and female Savannah Sparrow. Samples were obtained during the breeding seasons of 2015 to 2016 in Southern Alberta.*

|  | Female Chestnut-collared Longspur | | | Male Chestnut-collared Longspur | | | Male Baird’s Sparrow | | | Female Savannah Sparrow | | | Male Savannah Sparrow | | |
| --- | --- | --- | --- | --- | --- | --- | --- | --- | --- | --- | --- | --- | --- | --- | --- |
| *Predictor* | *Beta* | *SE* | *p* | *Beta* | *SE* | *p* | *Beta* | *SE* | *p* | *Beta* | *SE* | *p* | *Beta* | *SE* | *p* |
| Intercept | 25.86 | 7.60 | **0.01** | 10.37 | 4.61 | **0.03** | 145 | 29.9 | **<0.01** | -25.6 | 23.5 | 0.29 | 46.0 | 11.4 | **<0.01** |
| Distance from Oil Well | 0.55 | 0.99 | 0.58 | 0.14 | 0.77 | 0.85 | 3.25 | 4.22 | 0.446 | 6.88 | 3.56 | **0.07** | -1.61 | 2.00 | 0.42 |
| Distance from Road | -1.38 | 0.89 | 0.12 | 0.43 | 0.62 | 0.49 | -13.6 | 4.81 | **0.01** | -3.04 | 4.12 | 0.47 | 0.26 | 2.8 | 0.92 |
| Time of Day | - 0.02 | 0.01 | **0.05** |  |  |  | -0.09 | 0.03 | **0.01** |  |  |  |  |  |  |
| Basal CORT |  |  |  |  |  |  |  |  |  | 11.2 | 4.01 | **0.01** |  |  |  |
| n | 61 | | | 86 | | | 41 | | | 18 | | | 87 | | |
| R^2^ / adjusted R^2^ | 0.088 / 0.040 | | | 0.008 / -0.016 | | | 0.318 / 0.263 | | | 0.466 / 0.351 | | | 0.013 / -0.011 | | |

*Table S12. Influence of distance to oil well types and simulated screwpump noise, age, weight (g), and day, on basal corticosterone levels (Log (ng/ml)) in female and male Chestnut-collared Longspur, male Baird’s Sparrow, and male and female Savannah Sparrow, 2015 to 2016 in Southern Alberta. Distances from center of each site or infrastructure/ playback unit and are measured in Log(m).*

|  | Female Chestnut-collared Longspur | | | Male Chestnut-collared Longspur | | | Male Baird’s Sparrow | | | Female Savannah Sparrow | | | Male Savannah Sparrow | | |
| --- | --- | --- | --- | --- | --- | --- | --- | --- | --- | --- | --- | --- | --- | --- | --- |
| *Predictors* | *Beta* | *SE* | *p* | *Beta* | *SE* | *p* | *Beta* | *SE* | *p* | *Beta* | *SE* | *p* | *Beta* | *SE* | *p* |
| (Intercept) | -1.25 | 1.31 | 0.343 | 0.52 | 1.73 | 0.763 | 8.06 | 2.35 | **0.002** | 3.95 | 2.72 | 0.170 | 3.38 | 1.18 | **0.005** |
| Control | 0.39 | 0.24 | 0.114 | 0.06 | 0.31 | 0.854 | 0.42 | 0.16 | **0.014** | -0.33 | 0.48 | 0.502 | 0.27 | 0.17 | 0.122 |
| Silent | -0.28 | 0.87 | 0.750 | -0.15 | 0.58 | 0.792 | -0.18 | 1.04 | 0.862 |  |  |  | -0.68 | 0.34 | **0.047** |
| Simulated  Screwpump | -0.88 | 0.36 | **0.016** | -0.26 | 0.50 | 0.602 | -13.96 | 9.32 | 0.143 | 1.50 | 1.60 | 0.365 | -0.37 | 0.70 | 0.598 |
| Pumpjack | 0.92 | 0.54 | **0.095** | 0.12 | 0.55 | 0.833 |  |  |  |  |  |  | -0.23 | 0.25 | 0.371 |
| Screwpump | -0.58 | 0.37 | 0.122 | -0.00 | 0.39 | 0.992 | -0.38 | 0.35 | 0.275 | -0.37 | 1.16 | 0.753 | 0.04 | 0.31 | 0.899 |
| Age: ASY |  |  |  | 0.32 | 0.22 | 0.148 |  |  |  |  |  |  | 0.29 | 0.14 | **0.040** |
| Weight |  |  |  |  |  |  | -0.29 | 0.10 | **0.006** |  |  |  |  |  |  |
| Season Day |  |  |  |  |  |  | -0.02 | 0.01 | **0.002** |  |  |  | -0.02 | 0.00 | **<0.001** |
| Observations | 84 | | | 115 | | | 46 | | | 20 | | | 104 | | |
| R^2^ / adjusted R^2^ | 0.193 / 0.095 | | | 0.041 / -0.051 | | | 0.473 / 0.322 | | | 0.213 / -0.150 | | | 0.251 / 0.162 | | |

*ASY: After second year bird – a bird in its third calendar year since hatching, indicating a mature adult bird.* ‘*Pumpjack’ refers to propane-powered pumpjack oil wells that draw oil from underground using a vertical see-saw motion. ‘Screwpump’ refers to propane-powered screwpump oil wells, another type of oil well without the associated large movements of a pumpjack. ‘Simulated screwpump” is a high-fidelity experiential noise treatment, broadcasting the sound of a screwpump oil well 24h a day in field absent of oil infrastructure throughout the breeding season. ‘Silent’ treatments have the same fencing and build as the playback infrastructure, but do not broadcast noise. Controls are field with no infrastructure nor experimental playback manipulations.*

*Table S13. Influence of distance oil wells types and simulated screwpump noise, basal corticosterone level (Log), time of day, and day of season, on stress response corticosterone levels (ng/ml) in female and male Chestnut-collared Longspur, male Baird’s Sparrow, and male and female Savannah Sparrow. Samples were obtained during the breeding seasons of 2015 to 2016 in Southern Alberta. Distances from center of each site or infrastructure/ playback unit and are measured in Log(m).*

|  | Female Chestnut-collared Longspur | | | Male Chestnut-collared Longspur | | | Male Baird’s Sparrow | | | Female Savannah Sparrow | | | Male Savannah Sparrow | | |
| --- | --- | --- | --- | --- | --- | --- | --- | --- | --- | --- | --- | --- | --- | --- | --- |
| *Predictors* | *Beta* | *SE* | *p* | *Beta* | *SE* | *p* | *Beta* | *SE* | *p* | *Beta* | *SE* | *p* | *Beta* | *SE* | *p* |
| (Intercept) | 35.2 | 14.3 | **0.02** | 18.4 | 14.9 | 0.22 | 71.9 | 40.2 | 0.08 | 27.0 | 52.9 | 0.62 | 61.8 | 30.3 | **0.05** |
| Control | -2.11 | 2.41 | 0.38 | -0.70 | 2.66 | 0.79 | 1.63 | 5.51 | 0.76 | -3.30 | 8.88 | 0.71 | -3.45 | 4.24 | 0.41 |
| Silent | 7.20 | 9.90 | 0.46 | 2.98 | 5.11 | 0.561 | 18.6 | 34.4 | 0.59 |  |  |  | 6.10 | 8.49 | 0.47 |
| Simulated screwpump | 4.37 | 3.56 | 0.22 | -2.53 | 4.30 | 0.558 | -129 | 313. | 0.68 | 24.9 | 28.5 | 0.40 | 19.6 | 19.36 | 0.31 |
| Screwpump | 1.98 | 3.79 | 0.60 | 1.11 | 3.36 | 0.742 | 5.59 | 12.6 | 0.66 | 28.0 | 20.4 | 0.19 | 0.79 | 7.67 | 0.91 |
| Pumpjack | 6.62 | 5.45 | 0.23 | 0.45 | 4.66 | 0.92 |  |  |  |  |  |  | -10.6 | 6.16 | **0.09** |
| Basal corticosterone |  |  |  |  |  |  |  |  |  | 10.9 | 4.96 | **0.05** | 3.75 | 2.54 | 0.14 |
| Time of Day | -0.03 | 0.01 | **0.01** |  |  |  | -0.09 | 0.04 | **0.03** |  |  |  |  |  |  |
| Season Day |  |  |  |  |  |  |  |  |  |  |  |  | -0.08 | 0.11 | 0.464 |
| n | 81 | | | 111 | | | 45 | | | 19 | | | 102 | | |
| R^2^ / adjusted R^2^ | 0.140 / 0.017 | | | 0.051 / -0.033 | | | 0.248 / 0.054 | | | 0.542 / 0.251 | | | 0.174 / 0.073 | | |

‘*Pumpjack’ refers to propane-powered pumpjack oil wells that draw oil from underground using a vertical see-saw motion. ‘Screwpump’ refers to propane-powered screwpump oil wells, another type of oil well without the associated large movements of a pumpjack. ‘Simulated screwpump’ is a high-fidelity experiential noise treatment, broadcasting the sound of a screwpump oil well 24h a day in field absent of oil infrastructure throughout the breeding season. ‘Silent’ treatments have the same fencing and build as the playback infrastructure, but do not broadcast noise. Controls are field with no infrastructure nor experimental playback manipulations.*
